# Behavioral outcomes of preschool children with congenital heart disease and controls

**DOI:** 10.3389/fped.2026.1725994

**Published:** 2026-03-24

**Authors:** Andrew T. M. Chew, Alexandra F. Bonthrone, Mirthe E. M. van der Meijden, Zeyuan Sun, Kuberan Pushparajah, John Simpson, A. David Edwards, Serena J. Counsell, Chiara Nosarti

**Affiliations:** 1 Department of Early Life Imaging, School of Biomedical Engineering and Imaging Sciences, King’s College London, London, United Kingdom; 2 Department of Child and Adolescent Psychiatry, Institute of Psychiatry, Psychology and Neuroscience, King’s College London, London, United Kingdom; 3 Paediatric Cardiology Department, Evelina London Children’s Healthcare, London, United Kingdom

**Keywords:** behavior, congenital heart disease, home environment, inattention, outcome, preschool

## Abstract

**Introduction:**

Behavioral outcomes may be suboptimal in school-age children and adolescents with congenital heart disease (CHD). However, little is known about the behavioral outcomes of preschool children with CHD. This study aimed to compare behavioural outcomes in preschool children with CHD and controls, and to investigate the impact of a cognitively stimulating home environment on these outcomes.

**Methods:**

Cross-sectional case-control study based on parent-rated questionnaires assessing child behavior and cognitively stimulating opportunities in the home environment in 56 preschool children (4–6 years) with CHD and 215 control participants. Validated questionnaires were used to assess temperament (Child Behavior Questionnaire), autism traits (Social Communication Questionnaire), ADHD symptoms (ADHD-Rating Scale-IV), empathy (EmQue), and behavioral difficulties (Strengths and Difficulties Questionnaire). Higher scores on these measures indicate more severe difficulties. The Cognitively Stimulating Parenting Scale was used to assess the presence of cognitive stimulating opportunities in the home environment.

**Results:**

Univariate robust regression analyses showed that children with CHD compared to controls had higher levels of age-adjusted hyperactivity/impulsivity (B = −0.339, *p* = 0.032), hyperactivity/inattention (B = −0.390, *p* = 0.032) and peer relationship problems (B = −0.298, *p* = 0.045), after controlling for gestational age at birth, sex and neighborhood deprivation, with results surviving false discovery rate correction. We did not find any differences between children with CHD and controls in the other behavioral measures assessed. Group (CHD or control) significantly moderated the relationship between cognitively stimulating opportunities at home and selective behavioral outcomes: hyperactivity/impulsivity, inattention and peer problems. More cognitively stimulating opportunities at home were associated with more favorable behavioral outcomes in children with CHD (hyperactivity/impulsivity: B = −0.092, *p* < 0.001; hyperactivity/inattention: B = −0.088, *p* < 0.001; peer problems: B = −0.124, *p* < 0.001) but not in controls (hyperactivity/impulsivity: B = −0.005, *p* = 0.727; hyperactivity/inattention: B = −0.019, *p* = 0.225; peer problems: B = −0.002, *p* = 0.911).

**Conclusions:**

Compared to controls, and after adjusting for potential confounders, preschool children with CHD have more hyperactivity/impulsivity, inattention and peer relationship problems. Fewer behavioral problems were associated with a more cognitively stimulating home environment, highlighting this modifiable factor as a promising target for future longitudinal research.

## Introduction

Congenital heart disease (CHD) remains the most common congenital anomaly affecting approximately 1% of livebirths worldwide (1, 2). Children with CHD are at increased risk of neurodevelopmental impairments and behavioral difficulties, with severity increasing with disease complexity (3). As children enter preschool, they encounter environments that require greater independence and self-regulation; hence the frequency and severity of behavioral difficulties may become more apparent (4).

Previous studies have identified a wide range of suboptimal behavioral outcomes in school age children and adolescents with CHD, with a predominance of internalizing (such as anxiety, depression, withdrawal) over externalizing symptoms (such as impulsivity, hyperactivity, aggression) (5–8), poorer emotional control, problems with self-esteem and body image, social interactions, and social cognition (9–13). There is also increasing evidence that children with CHD are at greater risk of inattention or being diagnosed with attention deficit hyperactivity disorder (ADHD) (14–18). Autism diagnosis or traits are also increased in school-age children, adolescents and young adults with CHD (19–22).

The behavioral outcomes of preschool children with CHD are not as well researched as those of school-age children and adolescents (6, 23, 24). The preschool years are a time of rapid socioemotional development, marked by rapid changes in development which lay the foundation for future learning in school (25). The studies assessing behavioral differences in preschool children with CHD show conflicting results, although these are difficult to compare directly due to methodological differences. Some studies have reported no behavioral differences in preschool children with CHD compared to population norms or controls (26–32), while a few have reported significant differences in emotional problems or autism traits (33–35) and ADHD symptoms (34, 36).

The early family environment may exert long-term influences on children's neurodevelopment (37–40). For instance, we have previously shown that a cognitively stimulating environment was associated with neurocognitive development in toddlerhood and better executive function in preschool children with CHD (41, 42). However, little is known about the role of a stimulating home environment in shaping the behavioral outcomes of preschool children with CHD.

This study assessed the behavioral profile of preschool children with CHD, testing the hypothesis that they exhibit less optimal behavioral outcomes than control children. A second exploratory aim was to examine the association between cognitively stimulating parenting and children's behavioral outcomes.

## Methods

### Participants

Inclusion criteria for the CHD sample were children with critical or serious CHD who took part in the Congenital Heart Imaging Project (REC: 07/H0707/105) between September 2014 and January 2020 and who had surgery or intervention by cardiac catheterization within the first year of life. Critical CHD was defined as hypoplastic left heart syndrome, transposition of the great arteries (TGA), pulmonary atresia with intact ventricular septum, interruption of the aortic arch, and all infants requiring surgery within the first 28 days of life with the following conditions: coarctation of the aorta, aortic valve stenosis, pulmonary valve stenosis; tetralogy of Fallot (TOF), pulmonary atresia with ventricular septal defect, and total anomalous pulmonary venous connection. Serious CHD was defined as any cardiac lesion not defined as critical that requires cardiac catheterization or surgery before 1 year of age (43).

Inclusion criteria for the control sample were children who participated in the Developing Human Connectome Project (dHCP; REC: 14/LO/1169) (44) and whose parents had consented to be approached for further research studies. We contacted parents of dHCP participants who were projected to be the same age as participants with CHD when questionnaires were completed. Exclusion criteria for all participants were children born before 31 completed weeks of gestation.

Parents of both children with CHD and controls were contacted when their children were between four and six years of age. Parents were emailed the study information sheet and questionnaires were posted to those who agreed to participate and provided informed written consent.

### Behavioral assessments

Five parent-rated measures were used to comprehensively evaluate children's behavioral profile. The Children's Behavior Questionnaire – Very Short Form (CBQ-VSF) measured three temperamental traits: Surgency, Negative Affectivity and Effortful Control (45). The Social Communication Questionnaire (SCQ) (46) was used to assess autism traits; it consists of 40 yes/no items that are summed to produce a total score. ADHD symptoms were assessed using the ADHD Rating Scale-IV (47) which measured Inattention and Hyperactivity-Impulsivity scores. The Empathy Questionnaire (EmQue) assessed the first three levels of empathy in infants' and young children's behaviours (48): Emotional Contagion, Attention to Others' Feelings, and Prosocial Actions; it has 20 items. The Strengths and Difficulties Questionnaire (SDQ) was used to measure psychological attributes and identify potential difficulties as well as strengths (49), such as emotional, conduct, hyperactivity/inattention, peer relationship problems, and prosocial behavior. Higher scores on these measures indicate more severe difficulties. Further details of these measures are provided in the Methods of Supplementary File.

### Environmental factors

The Cognitively Stimulating Parenting Scale (CSPS) was used as a covariate in our models to measure the availability and variety of experiences that promote cognitive stimulation at home and in the family. The CSPS was adapted from the Home Observation Measurement of the Environment (41). Parents were asked if their children have access to child-size tables and chairs, and stimulating materials, such as storybooks, coloring books, musical instruments, and others. A copy of the CSPS is attached in the Appendix of the Supplementary File. We used the Index of Multiple Deprivation (IMD) as a measure of neighborhood deprivation, and a proxy for parental socio-economic status. The IMD combines information from 7 domains to produce an overall relative measure of neighbourhood deprivation in England. The 7 domains that make up the composite IMD are weighted differently: Income (22.5%), Employment (22.5%), Education/Skills (13.5%), Health/Disability (13.5%), Crime (9.3%), Housing/Services (9.3%), and Living Environment (9.3%). Parents' residential postcode at follow-up assessment was used to calculate the IMD from the 2015 data release and reported as percentile ranks (http://imd-by-postcode.opendatacommunities.org/; Accessed December 15, 2023).

### Statistical analysis

Data analysis was completed in R using RStudio 2023.12.0 Build 369 and IBM SPSS Statistics (version 29; IBM Corp.). Firstly, missing data (1.6%) were imputed in R using missForest (50). This method of imputation has the advantages that it has no need for tuning parameters nor does it require assumptions about distributional aspects of the data. Data were assessed for normality using density plots, QQ-plots, and Shapiro–Wilk tests. Variables were also checked for collinearity and homoscedacity. Demographic and environmental data of children with CHD and controls were compared using Mann–Whitney U test.

Data of all 14 behavioral variables from five behavioral measures, which are developmentally sensitive, were regressed on age at the time of parent questionnaire completion, and the resulting residuals were standardised (Z-scored). Thereafter, age-adjusted outcomes were compared between groups (CHD or control), using the robust regression function in SPSS. This procedure uses iteratively reweighted least squares with an M-estimation algorithm to reduce the influence of outliers and model assumption violations. Sex, GA at birth, and IMD were used as confounders in the analyses and statistical significance was determined after false discovery rate (FDR) correction (51).

As validated clinical cut-offs exist for ADHD-RS (52), SCQ (46) and SDQ (53), we also calculated the proportions of children with CHD and controls above clinical thresholds, with adjusted odds ratios to support clinical relevance. Clinical relevance was assessed using standardized effect sizes for continuous outcomes (Cohen’s *d*) and adjusted odds ratios for dichotomous outcomes, reported with 95% confidence intervals.

Using the PROCESS macro for SPSS (54), moderated multiple regression analyses were conducted to test group (CHD or control) x CSPS interactions for behavioral variables that showed significant group differences, covarying for sex, GA and IMD. CSPS scores were not age adjusted as they were conceptualized as an environmental characteristic, hence not expected to vary in an age-dependent way across our age range.

The following sensitivity analyses, presented in Supplementary File, were also conducted: 1) comparison of age-adjusted outcomes between groups (CHD or control) after removal of outlier data, defined as any variable more than 1.5 times of IQR below the first quartile, or more than 1.5 times of IQR above the third quartile (55); 2) comparison of age-adjusted outcomes between groups (CHD or control) after winsorization and 3) after removal of five CHD participants who had confirmed or suspected genetic abnormality.

## Results

Eighty-six parents of children with CHD agreed to receive questionnaires, and 66 were returned (77% return rate). Five children were excluded, as they were born at less than 31 weeks' gestation, and 5 children did not have cardiac surgery. Data from 56 children with CHD were included in the analysis. Five children in the CHD sample had confirmed or suspected genetic abnormality [2 children had CHARGE (coloboma, heart defects, choanal atresia, growth retardation, genital abnormalities, and ear abnormalities) syndrome, 2 had 22q11 deletion, and 1 child had a suspected but not confirmed genetic abnormality].

**Figure 1:**
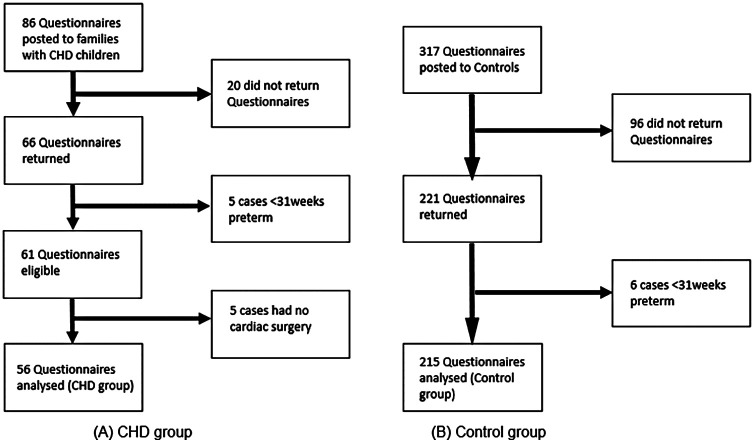
Flow chart of participant recruitment for both **(A)** CHD and **(B)** control groups.

In total, 317 parents of control children agreed to receive questionnaires and 221 were returned (70% return rate). Six children were born at less than 31 weeks' gestation. The final control sample size comprised 215 children. Figure 1 shows details of participant recruitment.

Table 1 shows participants’ demographic and environmental data, the primary cardiac diagnoses of the children with CHD, the number of CHD cases with cyanosis, and number of cardiac surgeries carried out. There was no difference between CHD and controls in sex distribution (*p* = 0.897), IMD (*p* = 0.756), or age at assessment (*p* = 0.921). Children with CHD were born at a younger gestational age (*p* < 0.001) and had lower CSPS scores (*p* < 0.001) compared to controls. Lower CSPS scores denote a less stimulating home environment. Although CSPS scores differed between CHD and control, score distributions were fairly similar (Supplementary Figure S1 in Supplementary File).

**Table 1:** Demographic, socio-environmental and clinical data of children with CHD and controls.

Variable name	CHD, *n* = 56	Controls, *n* = 215
Age at assessment, months (median, IQR)	50.53 (48.52–58.13)	49.62 (48.60–60.62)
Sex^a^, male (n, %)	28 (50.0)	103 (47.9)
Gestational age at birth, wks (median, IQR)	38.43 (37.89–38.86)	39.71 (37.71–40.71)
IMD quintile (n, %)		
1 (lowest)	7 (12.5)	35 (16.3)
2	13 (23.2)	57 (26.5)
3	11 (19.6)	48 (22.3)
4	12 (21.4)	36 (16.7)
5 (highest)	13 (23.2)	39 (18.1)
CSPS (median, IQR)	36.50 (31.00–38.25)	38.00 (36.00–40.00)
Cardiac physiology of CHD children (n, %)		
Transposition of great arteries	22 (39)	
Coarctation of the aorta	12 (21)	
Tetralogy of Fallot	11 (20)	
Pulmonary stenosis	4 (7)	
Pulmonary atresia	3 (5)	
Truncus arteriosus	1 (2)	
Hypoplastic Left Heart Syndrome	1 (2)	
Aortic stenosis	1 (2)	
Tricuspid atresia	1 (2)	
Cyanotic	31 (55)	
Antenatal diagnosis of CHD	55 (98)	

^a^ Assigned sex at birth.

Results of univariate robust regression of age-adjusted outcomes controlling for sex, GA and IMD showed that children with CHD had higher hyperactivity/impulsivity (ADHD-RS), hyperactivity/inattention scores (SDQ) and more peer relationship problems (SDQ) compared to controls, all with moderately large effect sizes (Table 2). Lower GA at birth was associated with higher hyperactivity/impulsivity scores (B = −0.056, *p* = 0.011), while girls had better effortful control scores than boys (B = 0.518, *p* < 0.001).

**Table 2:** Behavioral outcome scores of children with CHD and controls.

Behavioral outcomes (*n*, median, IQR)	CHD (*n* = 56)	Control (*n* = 215)	B coefficient	p_FDR_ value	Cohen’s *d* effect size (95% confidence interval)
Surgency (CBQ)	55.0 (44.5–60.0)	54.0 (47.0–60.0)	−0.042	0.395	−0.038 (−0.332, 0.257)
Negative Affect (CBQ)	48.0 (40.0–55.0)	46.0 (39.0–54.0)	0.112	0.269	0.161 (−0.134, 0.455)
Effortful Control (CBQ)	64.0 (57.8–68.5)	65.0 (60.0–69.0)	−0.136	0.209	−0.171 (−0.465, 0.124)
Inattention (ADHD-RS)	5.0 (3.0–8.0)	4.0 (1.0–7.0)	0.191	0.119	0.287 (−0.008. 0.582)
Hyperactivity/impulsivity (ADHD-RS)	5.8 (4.0–12.0)	5.0 (2.0–7.0)	0.339	0.032*	0.403 (0.107, 0.699)
Social communication (SCQ)	5.5 (2.8–10.0)	4.0 (2.0–7.0)	0.282	0.071	0.302 (0.106, 0.598)
Emotion Contagion (EmQue)	2.0 (0.0–4.0)	1.0 (0.0–3.0)	0.214	0.119	0.188 (−0.106, 0.483)
ATOF (EmQue)	9.0 (7.0–10.0)	9.0 (8.0–11.0)	−0.228	0.119	−0.223 (−0.517, 0.072)
Prosocial Actions (EmQue)	6.0 (5.0–8.0)	6.0 (5.0–8.0)	−0.105	0.269	−0.137 (−0.431, 0.157)
Emotional problems (SDQ)	1.0 (0.0–3.0)	1.0 (0.0–2.0)	0.255	0.071	0.250 (−0.045, 0.545)
Conduct problems (SDQ)	2.0 (1.0–3.0)	1.0 (0.0–2.0)	0.206	0.122	0.226 (−0.068, 0.521)
Hyperactivity/inattention (SDQ)	4.0 (2.3–6.8)	3.0 (1.0–5.0)	0.390	0.032*	0.405 (0.109, 0.701)
Peer relationship problems (SDQ)	1.0 (0.0–3.8)	1.0 (0.0–2.0)	0.298	0.045*	0.403 (0.107, 0.699)
Prosocial behavior (SDQ)	7.0 (6.0–9.0)	8.0 (6.0–9.0)	−0.213	0.122	−0.292 (−0.587, 0.004)

Descriptive statistics for behavioral outcome scores in children with CHD and controls are presented using raw data, whereas group comparisons derived from robust regression models are based on age-adjusted standardized scores.

*p < 0.05 after FDR correction, controlling for GA, sex and IMD.

Results of sensitivity analyses, presented in Supplementary File, showed that removal of outlier data (listed in Supplementary Table S1) did not change the observed differences between children with CHD and controls observed in the full sample. These are presented in Supplementary Table S2. After winsorization, comparison of age-adjusted outcomes between groups (CHD or control) revealed two additional findings, children with CHD displayed higher socio-communication (SCQ) and emotional (SDQ) problems compared to controls (Supplementary Table S3). After removal of five CHD participants with confirmed or suspected genetic abnormality, the significant group differences in ADHD traits observed in the full sample persisted, while differences in peer problems (SDQ) were no longer significant (Supplementary Table S4).

Table 3 shows the proportions of participants scoring above established clinical thresholds for the ADHD-RS, SCQ and SDQ. Relative to controls, children with CHD had significantly higher odds of clinically significant ADHD and autism traits and peer relationship problems.

**Table 3:** Clinically significant scores in children with CHD and controls.

Behavioral outcomes	CHD cases above cutoff *n* (%)	Control cases above cutoff *n* (%)	*P* value	aOR (95% confidence interval)
Inattention (ADHD-RS)	7 (12.5)	7 (3.3)	0.006*	0.208 (0.067, 0.644)
Hyperactivity/impulsivity (ADHD-RS)	6 (10.7)	9 (4.2)	0.045*	0.318 (0.104, 0.975)
Social communication (SCQ)	4 (7.1)	3 (1.4)	0.022*	0.155 (0.032, 0.763)
Emotional (SDQ)	3 (5.4)	13 (6.0)	0.876	1.108 (0.303, 4.060)
Conduct (SDQ)	11 (19.6)	25 (11.6)	0.140	0.547 (0.246, 1.217)
Hyperactivity/inattention (SDQ)	14 (25.0)	24 (11.2)	0.007*	0.351 (0.164, 0.749)
Peer problems (SDQ)	14 (25.0)	18 (8.4)	0.001*	0.267 (0.121, 0.589)
Prosocial (SDQ)	7 (12.5)	16 (7.4)	0.222	0.545 (0.206, 1.444)

*p < 0.05; aOR, adjusted OR, controlling for GA, sex and IMD.

**Figure 2:**
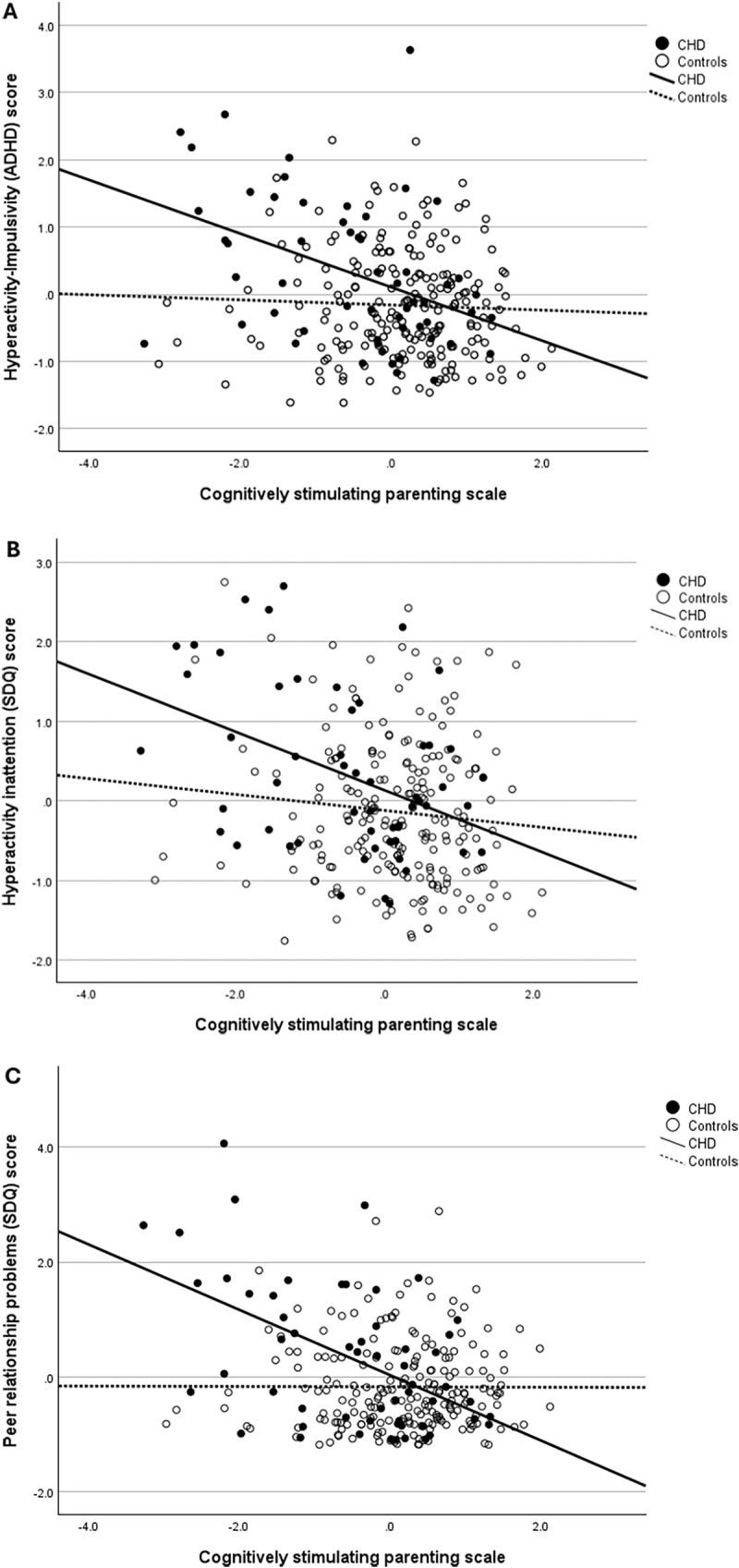
Cognitively stimulating parenting scale (CSPS) scores were significantly associated with **(A)** hyperactivity/impulsivity (ADHD) scores in the CHD group but not in controls, **(B)** hyperactivity/inattention (SDQ) scores in the CHD group but not in controls, **(C)** peer relationship problem (SDQ) scores in the CHD group but not in controls.

A significant group interaction (CHD or control) was observed in the relationship between CSPS scores and hyperactivity/impulsivity (ADHD) (*p* = 0.001), hyperactivity/inattention (SDQ) (*p* = 0.018), and peer relationship problems (SDQ) (*p* < 0.001). Higher CSPS scores correlated with lower levels of hyperactivity/impulsivity (ADHD-RS) (B = −0.092, *p* < 0.001), hyperactivity/inattention (SDQ) (B = −0.088, *p* < 0.001), peer relationship problems (SDQ) (B = −0.124, *p* < 0.001) in children with CHD, but not in controls [hyperactivity/impulsivity (ADHD): B = −0.005, *p* = 0.727; hyperactivity/inattention (SDQ): B = −0.019, *p* = 0.225; peer relationship problems (SDQ): B = −0.002, *p* = 0.911] (Figures 2A–C).

## Discussion

Our results demonstrated that preschool children with CHD exhibit more hyperactivity-impulsivity and inattention problems, and peer relationship problems than control children. We did not find any differences between children with CHD and controls in the other behavioral measures assessed. Furthermore, we found that a more cognitively stimulating home environment was associated with lower hyperactivity-impulsivity, inattention and peer relationship problems in preschool children with CHD but not in controls.

Studies investigating the prevalence of ADHD and the severity of ADHD symptoms in CHD have largely been conducted in school-aged children, adolescents and adults (23). The prevalence of ADHD traits or diagnosis is higher in cohorts of children and adolescents with mixed types of CHD (56–61) and in single physiology groups (HLHS, d-TGA, TOF) (3, 14, 16) when compared to healthy controls or population norms. Multiple studies have shown that cyanotic CHD is a significant risk factor for ADHD diagnosis or traits (62–65). However, there are only a few studies investigating ADHD traits in preschool children with CHD. A small case-control study of 12 preschool children with TGA and 30 controls did not show any between-group difference in level of hyperactivity/inattention (33). Our results showing higher prevalence of hyperactivity, impulsivity and inattention behaviours in preschool children with CHD compared to contemporaneous controls are in line with two studies comparing ADHD traits in preschool children with CHD and population norms (34, 36). Our previous work, using a comprehensive eye tracking battery to assess visual attention in toddlers (22 months) with CHD and controls, showed that toddlers with CHD had slower reaction times during selective and exogenous attention tasks (66), which suggests that precursors of inattention and behavioral control difficulties could already be observed in toddlerhood. Although our results show that preschool children with CHD have higher hyperactivity, impulsivity and inattention traits (Table 2), the majority of children have scores that fall below clinical cutoffs, hence do not meet the threshold for a clinical diagnosis.

We also found that our cohort of preschool children with CHD had more parent-rated peer relationship problems than controls. These findings are in line with previous studies that have shown young adults with atrial or ventricular septal defects have a fourfold increased risk of social interaction difficulties compared to healthy peers (67). School-age children with CHD had previously been shown to experience peer relationship problems using the same measure we used (61). Peer relationship problems may be partly driven by impaired capacity to recognize facial emotion expressions and identify false beliefs (Theory of Mind), which have previously been reported in school-aged children with CHD (19, 68, 69). There is increasing interest in understanding early childhood peer relationships, as poor relationships may negatively impact preschool attendance (25), physical activity (70) and later mental health (71).

Sensitivity analyses largely supported the robustness of the primary findings. Removal of outliers did not meaningfully alter group differences in hyperactivity/impulsivity, inattention, or peer relationship problems, indicating that these effects were not driven by extreme scores.

Peer relationship problems were no longer significant after excluding children with confirmed or suspected genetic abnormalities, whereas ADHD-related differences remained significant. This pattern suggests that attentional difficulties may represent a more consistent feature of developmental risk in CHD, while socio-emotional outcomes appear more heterogeneous and potentially influenced by co-occurring genetic vulnerability.

Together, these analyses strengthen confidence in the stability of ADHD-related findings while highlighting variability in socio-emotional domains within the CHD population.

In our study we did not find differences in empathy or temperament in preschool children with CHD and no previous studies have measured these outcomes in a similar age group. In the full sample we also found no difference in autism traits between CHD preschoolers and controls when assessed using a screening tool (SCQ). However, results of sensitivity analyses after winsorization identified elevated autism traits (i.e., social communication) and emotional difficulties in the CHD group, suggesting that subtle distributional characteristics may influence detection of socio-emotional differences. Previous studies using the SCQ (35) and the Child Behavior Checklist (CBCL) (34) reported that preschool children with CHD were more likely to screen for autism and pervasive developmental disorder when compared to population norms, respectively. Therefore, these findings warrant replication in larger samples of preschool children with CHD.

This study provides further evidence of the role of a cognitively stimulating home environment in affecting outcomes of at-risk groups of children. We have previously shown that a more cognitively stimulating home environment was associated with greater cognitive scores on the Bayley Scales of Infant and Toddler Development at 22 months in toddlers with CHD (41), and better executive function outcomes in our preschool cohort with CHD (42). In this study, a more cognitively stimulating home environment was associated with lower hyperactivity-impulsivity, inattention and peer relationship problems in children with CHD but not in controls. This pattern suggests that an enriched home environment may exert a protective, and importantly modifiable, influence that promotes resilience in this population. The absence of a comparable effect in controls further indicates that children with CHD may be differentially susceptible to their environment (72). Such susceptibility may reflect heightened neuroplasticity, whereby developmental outcomes are more sensitive to environmental conditions and can be shaped in either adaptive or maladaptive directions depending on the child's context (73). Furthermore, genetic factors may contribute to this variability, as polymorphisms associated with CHD, including variants of the ApoE gene, have been associated with behavioral problems (34).

### Strengths and limitations

A strength of this study is the inclusion of a large contemporaneous control sample. In addition, the age range of our cohort is fairly narrow within their preschool years, thus not affected by high levels of teacher-led schooling, which modulates behavior and socioemotional development (74).

There are several limitations in this study. Our sample is from a single centre and may not be representative of the UK, or other cultures, especially where there are significant differences in how preschool children are raised (75). The use of parent-rated questionnaires raises the possibility that common method variance may have contributed to the observed associations, potentially inflating relationships among variables. Additional limitations inherent to parent-reported measures include possible misunderstanding or misinterpretation of questionnaire items, recall bias stemming from prior traumatic experiences or recent hospitalizations involving their children, respondent fatigue, and systematic response biases associated with parenting a child with CHD. Future studies should compare data from parent-rated questionnaires to those obtained with objective assessments (76). Although the measures we used, such as the ADHD-RS, have robust psychometric properties and are widely used to screen for ADHD symptoms and correlate with clinical diagnoses (52), they do not replace comprehensive diagnostic evaluations that incorporate clinical judgment, cross-setting impairment, and developmental history. While we adjusted for neighborhood deprivation (IMD), important family-level factors not captured by the IMD, including parental education, family structure, and parental mental health, were not measured and may confound the observed associations. Notably, CSPS is likely correlated with dimensions of socioeconomic status and parental wellbeing (77). Furthermore, it remains unclear whether cognitively stimulating environments influence child behaviors, or whether child behavior, in turn, affects the degree of cognitive stimulation provided by caregivers. Parental mental health issues such as anxiety and depression may impact a child's functioning through shared genetic vulnerability and parenting environment (78). Additional reporting bias may be further introduced into parent-rated questionnaires (79). A final limitation is our cross-sectional design, which did not allow us to investigate whether any significant behavioral difficulties in the preschool years are associated with future psychopathology.

## Conclusion

We have shown that preschool children with CHD have more hyperactivity-impulsivity, inattention and peer relationship problems compared to healthy controls. Clinically, these findings highlight the value of routinely monitoring behavioral outcomes in preschool children with CHD. Incorporating brief screening and early referral into standard follow-up care may support earlier identification and management of emerging difficulties, alongside ongoing cardiac care. Our study further showed that higher levels of cognitive stimulation in the home environment were significantly associated with fewer behavioral problems. Thus environmental factors should be considered when planning intervention research aimed at improving behavioral outcomes in children with CHD. Supporting parents of preschool children with CHD to foster a stimulating home learning environment may be particularly important, given the growing evidence for the effectiveness of early home-based interventions (80, 81).

## Data Availability

The datasets presented in this article are not readily available because there was no consent taken from parents for data to be shared. Requests to access the datasets should be directed to serena.counsell@kcl.ac.uk.
