# Supplementary material for: Behavioral outcomes of preschool children with congenital heart disease and controls
Source: Front Pediatr. 2026 Mar 24;14:1725994. doi: 10.3389/fped.2026.1725994 (PMC13054659; doi:10.3389/fped.2026.1725994)
Supplement: Supplementary file 1 [file Supplementaryfile1.docx]

**Supplementary File**

**Method**

Details of the questionnaires used are described below.

**1, Children’s Behavior Questionnaire—Very Short Form (CBQ-VSF)**
The Children’s Behavior Questionnaire—Very Short Form (CBQ-VSF) is a 36-item parent-rated questionnaire assessing temperament, which is validated for children aged 3–8 (1). The CBQ is based on Rothbart’s psychobiological theory of temperament, which defined temperament as relatively stable and biologically based individual differences in levels of reactivity and self-regulation (2). This questionnaire asked parents to describe their children in situations occurring in everyday life using a 7-point scale from 1 (extremely untrue of your child) to 7 (extremely true of your child); thus higher scores indicate higher levels of a temperamental trait. The CBQ-VSF provides scores for three temperamental traits: Surgency, Negative Affectivity and Effortful Control. Surgency is characterised by a disposition toward positive emotions, high activity level and a rapid approach to potential rewards (3). Negative Affectivity is characterised by shyness, discomfort, anger–frustration, fear, sadness and un-soothability. Effortful Control encompasses voluntary attentional focusing, attentional shifting, inhibitory and activational control of behaviours (4).

**2, Attention-Deficit Hyperactivity Disorder Rating Scale IV (ADHD-RS IV)**

The ADHD RS-IV (5) scale consists of 18 ADHD symptoms as defined in the DSM-IV, to be rated on a four-point Likert scale ranging from 0 (the symptom is “never/rarely” present) to 3 (the symptom is “very often” present). The scale is designed to generate an Inattentive Score (0–27 points on odd numbered items), a Hyperactive/ Impulsive Score (0–27 points on even numbered items), and a Total Score (0–54 points on all items) (6). It has been translated into many languages and remains valid for assessing the severity of ADHD symptoms in children and adolescents (7) and longitudinally (8), and in those with other clinical conditions (9, 10) or after interventions (11).

**3, The Social Communication Questionnaire (SCQ)**

The Social Communication Questionnaire (12) is an autism screener which has 40-item parent-completed Yes/No questions. The SCQ was developed based on the established parental interview, the autism diagnostic interview (ADI) (13) and DSM-IV. The questionnaire can be used to evaluate anyone over age 4.0, as long as his or her mental age exceeds 2.0 years (14). The SCQ has been used in research and clinical settings, mainly in high-income settings (15-17). Recently a few studies have shown the validity of the SCQ in more rural settings in lower- and middle-income countries (18-21) . As it takes approximately 10 min to answer, the SCQ is a cost-effective way to determine whether an individual should be referred for a complete diagnostic evaluation.

**4, Empathy Questionnaire (EmQue)**

EmQue is a 20-item questionnaire aimed at observing the first three levels of empathy in infants’ and young children’s behaviours (22): Emotional Contagion, Attention to Others’ Feelings, and Prosocial Actions.

Hoffman had described the emotional development of children in four levels (23) and the EmQue measures the first three levels. ‘Emotion Contagion’ (24) manifests within the first year of life where infants attend to others’ emotions. However witnessing someone in distress may result in a similar affective response due to automatic imitation (25). Infants this young cannot yet differentiate between self and other, and act as though what happened to the other person happened to them (26). ‘Attention to Others’ Feelings’ is assumed to start at about one year of age. Infants become aware that although they feel distressed, it is not oneself but someone else who is in actual danger or pain. They become more aware of other people’s emotions, and direct their attention to affective displays and concern of others. At ‘Prosocial Actions’ level, children become more responsive to others’ emotional displays, and start to react prosocially. This ability to intervene on behalf of others during the second year of life can take a variety of forms, including helping, sharing, and comforting (27).

**5, Strength and Difficulties Questionnaire**

The SDQ is a brief behavioral screening questionnaire for children and adolescents aged 2 to 17 years (28). Designed to measure psychological attributes and identify potential difficulties as well as strengths, the SDQ consists of 25 items divided into five subscales: Emotional Symptoms Scale, Conduct Problems, Hyperactivity/Inattention, Peer Relationship Problems, and Prosocial Behavior Scale. Each subscale has five items, and respondents rate each item on a three-point scale: "Not True," "Somewhat True," or "Certainly True." The scores for each subscale can be summed to provide a total difficulties score, and individual scores can help identify specific areas of concern. In community samples, multi-informant SDQs can predict the presence of a psychiatric disorder with good specificity and moderate sensitivity (29, 30). The SDQ is freely available and being used as a research tool throughout the world in developmental, genetic, social, clinical and educational studies, and has been translated into over 80 languages. We used the Parent Version to collect data from parent’s perspective.

**6, The Cognitively Stimulating Parenting Scale (CSPS)**

The CSPS (31-33) is a 28-item questionnaire adaptation of the Home Observation for Measurement of the Environment Inventory (34) that is completed by parents.

The CSPS assesses the availability and variety of experiences that promote cognitive stimulation at home and in the family. This includes availability of educational toys, parental interactions such as teaching words or reading stories, and cognitively stimulating activities such as family excursions or trips (31). It is attached in the Appendix below.

**Figure S1: Distribution of CSPS scores for both groups (CHD, control)**

Both groups have fairly similar spread of results.


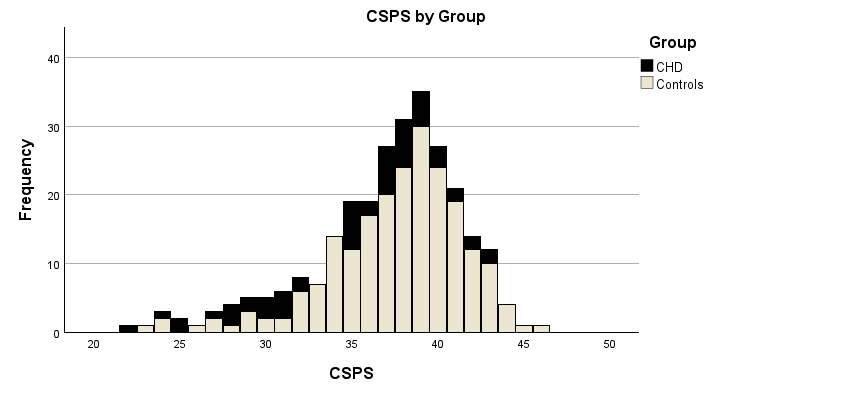


**Table S1: Number of outliers removed for each variable, and sample size used in analyses**

| **Outcomes** | **CHD, Total n=56** | **Controls, Total n=215** |
| --- | --- | --- |
| Surgency (CBQ) | 0 (n=56) | 1 (n=214) |
| Negative Affect (CBQ) | 1 (n=55) | 0 (n=215) |
| Effortful Control (CBQ) | 2 (n=54) | 6 (n=209) |
| Inattention (ADHD-RS) | 2 (n=54) | 5 (n=210) |
| Hyperactivity-impulsivity (ADHD-RS) | 0 (n=56) | 6 (n=209) |
| Social communication (SCQ) | 2 (n=54) | 3 (n=212) |
| Emotion Contagion (EmQue) | 0 (n=56) | 7 (n=208) |
| Attention to Others’ Feelings (EmQue) | 1 (n=55) | 5 (n=210) |
| Prosocial Actions (EmQue) | 1 (n=55) | 0 (n=215) |
| Emotional (SDQ) | 1 (n=55) | 12 (n=203) |
| Conduct (SDQ) | 0 (n=56) | 4 (n=211) |
| Hyperactivity/inattention (SDQ) | 0 (n=56) | 4 (n=211) |
| Peer problems (SDQ) | 0 (n=56) | 7 (n=208) |
| Prosocial (SDQ) | 1 (n=55) | 1 (n=214) |

**Table S2: Age-adjusted behavioral outcomes of children with CHD and controls after removal of outliers.**

| **Outcomes (median, IQR)** | **CHD** | **Controls** | **B coefficient** | **pFDR** |
| --- | --- | --- | --- | --- |
| Surgency (CBQ) | 55.0 (44.5 - 60.0) | 54.0 (47.0 - 60.0) | 0.048 | 0.380 |
| Negative Affect (CBQ) | 48.0 (40.0 - 55.0) | 46.0 (39.0 - 64.0) | -0.135 | 0.221 |
| Effortful Control (CBQ) | 64.0 (57.8 - 68.5) | 65.0 (60.0 - 69.0) | 0.122 | 0.221 |
| Inattention (ADHD-RS) | 5.0 (3.0 - 8.0) | 4.0 (1.0 - 7.0) | -0.182 | 0.118 |
| Hyperactivity-impulsivity (ADHD-RS) | 5.8 (4.0 - 12.0) | 5.0 (2.0 - 7.0) | -0.349 | 0.030* |
| Social communication (SCQ) | 5.5 (2.8 - 10.0) | 4.0 (2.0 - 7.0) | -0.290 | 0.052 |
| Emotion Contagion (EmQue) | 2.0 (0 - 4.0) | 1.0 (0 - 3.0) | -0.223 | 0.094 |
| Attention to Others’ Feelings (EmQue) | 9.0 (7.0 - 10.0) | 9.0 (8.0 - 11.) | 0.246 | 0.094 |
| Prosocial Actions (EmQue) | 6.0 (5.0 - 8.0) | 6.0 (5.0 - 8.0) | 0.072 | 0.346 |
| Emotional (SDQ) | 1.0 (0 - 3.0) | 1.0 (0 - 2.0) | -0.262 | 0.067 |
| Conduct (SDQ) | 2.0 (1.0 - 3.0) | 1.0 (0 - 2.0) | -0.226 | 0.109 |
| Hyperactivity/inattention (SDQ) | 4.0 (2.3 - 6.8) | 3.0 (1.0 - 5.0) | -0.410 | 0.030* |
| Peer problems (SDQ) | 1.0 (0 - 3.8) | 1.0 (0 - 2.0) | -0.305 | 0.042* |
| Prosocial (SDQ) | 7.0 (6.0 - 9.0) | 8.0 (6.0 - 9.0) | 0.194 | 0.142 |

Outliers defined as more than 1.5 times of IQR below 1st quartile or above 3rd quartile; *p<0.05

**Table S3: Age-adjusted behavioral outcomes of children with CHD and controls after winsorization at 5^th^ and 95^th^ centiles.**

| **Outcomes (median, IQR)** | **CHD** | **Controls** | **B coefficient** | **pFDR** |
| --- | --- | --- | --- | --- |
| Surgency (CBQ) | 55.0 (44.5 - 60.0) | 54.0 (47.0 - 60.0) | 0.038 | 0.401 |
| Negative Affect (CBQ) | 47.5 (40.0 - 55.0) | 46.0 (39.0 - 54.0) | -0.085 | 0.305 |
| Effortful Control (CBQ) | 64.0 (57.0 - 68.0) | 64.0 (59.0 - 69.0) | 0.147 | 0.177 |
| Inattention (ADHD-RS) | 5.0 (3.0 - 8.0) | 4.0 (1.0 - 7.0) | -0.212 | 0.114 |
| Hyperactivity-impulsivity (ADHD-RS) | 5.8 (4.0 - 12.0) | 5.0 (2.0 - 8.0) | -0.352 | 0.037* |
| Social communication (SCQ) | 6.0 (3.0 - 10.8) | 4.0 (2.0 - 7.0) | -0.324 | 0.038* |
| Emotion Contagion (EmQue) | 2.0 (0 - 4.0) | 1.0 (0 - 3.0) | -0.204 | 0.114 |
| Attention to Others’ Feelings (EmQue) | 9.0 (7.0 - 10.0) | 9.0 (8.0 - 11.0) | 0.225 | 0.114 |
| Prosocial Actions (EmQue) | 6.0 (4.3 - 7.8) | 6.0 (5.0 - 8.0) | 0.098 | 0.300 |
| Emotional (SDQ) | 1.0 (0 - 3.0) | 1.0 (0 - 2.0) | -0.291 | 0.038* |
| Conduct (SDQ) | 2.0 (1.0 - 3.0) | 2.0 (1.0 - 2.0) | -0.206 | 0.116 |
| Hyperactivity/inattention (SDQ) | 4.0 (2.3 - 6.8) | 3.0 (1.0 - 5.0) | -0.387 | 0.036* |
| Peer problems (SDQ) | 1.0 (0 - 3.8) | 1.0 (0 - 2.0) | -0.317 | 0.040* |
| Prosocial (SDQ) | 7.0 (5.3 - 9.0) | 8.0 (6.0 - 9.0) | 0.217 | 0.114 |
| *p<0.05 |  |  |  |  |

**Table S4: Age-adjusted behavioral outcomes of children with CHD and controls after removal of cases with confirmed or suspected genetic abnormality.**

| **Outcomes (median, IQR)** | **CHD (n=51)** | **Control (n=215)** | **B coefficient** | **pFDR** |
| --- | --- | --- | --- | --- |
| Surgency (CBQ) | 55.0 46.0 - 60.0) | 54.0 (47.0 - 60.0) | -0.005 | 0.488 |
| Negative Affect (CBQ) | 48.0 (40.0 - 55.0) | 46.0 (39.0 - 54.0) | -0.133 | 0.281 |
| Effortful Control (CBQ) | 64.0 (57.0 - 70.0) | 64.0 (59.0 - 69.0) | 0.079 | 0.341 |
| Inattention (ADHD-RS) | 5.0 (3.0 - 8.0) | 4.0 (1.0 - 7.0) | -0.218 | 0.140 |
| Hyperactivity-impulsivity (ADHD-RS) | 6.0 (4.0 - 12.0) | 5.0 (2.0 - 8.0) | -0.388 | 0.045* |
| Social communication (SCQ) | 6.0 (3.0 - 10.0) | 4.0 (2.0 - 7.0) | -0.295 | 0.072 |
| Emotion Contagion (EmQue) | 2.0 (0 - 4.0) | 1.0 (0 - 3.0) | -0.220 | 0.140 |
| Attention to Others’ Feelings (EmQue) | 9.0 (7.0 - 11.0) | 9.0 (8.0 - 11.0) | 0.132 | 0.281 |
| Prosocial Actions (EmQue) | 6.0 (5.0 - 8.0) | 6.0 (5.0 - 8.0) | -0.018 | 0.488 |
| Emotional (SDQ) | 1.0 (0 - 3.0) | 1.0 (0 - 2.0) | -0.265 | 0.072 |
| Conduct (SDQ) | 2.0 (1.0 - 3.0) | 2.0 (1.0 - 2.0) | -0.166 | 0.253 |
| Hyperactivity/inattention (SDQ) | 4.0 (2.0 - 7.0) | 3.0 (1.0 - 5.0) | -0.376 | 0.046* |
| Peer problems (SDQ) | 1.0 (0 - 3.0) | 1.0 (0 - 2.0) | -0.197 | 0.140 |
| Prosocial (SDQ) | 7.0 (6.0 - 9.0) | 8.0 (6.0 - 9.0) | 0.094 | 0.341 |

*p<0.05

**References:**

1. Putnam SP, Rothbart MK. Development of short and very short forms of the Children's Behavior Questionnaire. Journal of personality assessment. 2006;87(1):102-12.

2. Derryberry D, Rothbart MK. Arousal, affect, and attention as components of temperament. Journal of personality and social psychology. 1988;55(6):958.

3. Putnam SP, Ellis LK, Rothbart MK. The structure of temperament from infancy through adolescence. In: Angleitner AEA, editor. Advances in research on temperament. 1652001. p. 163-80.

4. Rothbart MK, Ahadi SA, Hershey KL, Fisher P. Investigations of temperament at three to seven years: The Children's Behavior Questionnaire. Child development. 2001;72(5):1394-408.

5. DuPaul GJ, Anastopoulos AD, Power TJ, Reid R, Ikeda MJ, McGoey KE. Parent ratings of attention-deficit/hyperactivity disorder symptoms: Factor structure and normative data. Journal of Psychopathology and Behavioral Assessment. 1998;20:83-102.

6. DuPaul GJ, Power TJ, Anastopoulos AD, Reid R. ADHD Rating Scale—IV: Checklists, norms, and clinical interpretation: The Guilford Press; 1998.

7. Zhang S, Faries D, Vowles M, Michelson D. ADHD rating scale IV: psychometric properties from a multinational study as clinician‐administered instrument. International journal of methods in psychiatric research. 2005;14(4):186-201.

8. Skogli EW, Orm S, Fossum IN, Andersen PN, Øie MG. Attention-deficit/hyperactivity disorder persistence from childhood into young adult age: a 10-year longitudinal study. Cognitive Neuropsychiatry. 2022;27(6):447-57.

9. Wyrwich KW, Auguste P, Yu R, Zhang C, Dewees B, Winslow B, et al. Evaluation of neuropsychiatric function in phenylketonuria: psychometric properties of the ADHD rating scale-IV and adult ADHD self-report scale inattention subscale in phenylketonuria. Value in Health. 2015;18(4):404-12.

10. Montagna A, Karolis V, Batalle D, Counsell S, Rutherford M, Arulkumaran S, et al. ADHD symptoms and their neurodevelopmental correlates in children born very preterm. PLoS One. 2020;15(3):e0224343.

11. Dölp A, Schneider-Momm K, Heiser P, Clement C, Rauh R, Clement H-W, et al. Oligoantigenic diet improves children’s ADHD rating scale scores reliably in added video-rating. Frontiers in psychiatry. 2020;11:730.

12. Rutter M, Bailey A, Lord C. Social communication questionnaire2003.

13. Lord C, Rutter M, Le Couteur A. Autism Diagnostic Interview-Revised: a revised version of a diagnostic interview for caregivers of individuals with possible pervasive developmental disorders. Journal of autism and developmental disorders. 1994;24(5):659-85.

14. Berument SK, Rutter M, Lord C, Pickles A, Bailey A. Autism screening questionnaire: diagnostic validity. The British Journal of Psychiatry. 1999;175(5):444-51.

15. Chandler S, Charman T, Baird G, Simonoff E, Loucas T, Meldrum D, et al. Validation of the social communication questionnaire in a population cohort of children with autism spectrum disorders. Journal of the American Academy of Child & Adolescent Psychiatry. 2007;46(10):1324-32.

16. Eaves LC, Wingert HD, Ho HH, Mickelson EC. Screening for autism spectrum disorders with the social communication questionnaire. Journal of Developmental & Behavioral Pediatrics. 2006;27(2):S95-S103.

17. Gau SS-F, Lee C-M, Lai M-C, Chiu Y-N, Huang Y-F, Kao J-D, et al. Psychometric properties of the Chinese version of the Social Communication Questionnaire. Research in Autism Spectrum Disorders. 2011;5(2):809-18.

18. Kipkemoi P, Savage JE, Gona J, Rimba K, Kombe M, Mwangi P, et al. Evaluation of the Psychometric Properties of the Social Communication Questionnaire in Rural Kenya. Journal of Autism and Developmental Disorders. 2025;55(8):2919-37.

19. Sangare M, Toure HB, Toure A, Karembe A, Dolo H, Coulibaly YI, et al. Validation of two parent-reported autism spectrum disorders screening tools M-CHAT-R and SCQ in Bamako, Mali. ENeurologicalSci. 2019;15:100188.

20. Ruparelia K, Abubakar A, Badoe E, Bakare M, Visser K, Chugani DC, et al. Autism spectrum disorders in Africa: current challenges in identification, assessment, and treatment: a report on the International Child Neurology Association Meeting on ASD in Africa, Ghana, April 3-5, 2014. Journal of child neurology. 2016;31(8):1018-26.

21. Nwokolo EU, Murphy GH, Langdon PE. Validation of the social communication questionnaire amongst Nigerian adolescents. Autism Research. 2024;17(1):66-77.

22. Rieffe C, Ketelaar L, Wiefferink CH. Assessing empathy in young children: Construction and validation of an Empathy Questionnaire (EmQue). Personality and individual differences. 2010;49(5):362-7.

23. Hoffman ML. Empathy and justice motivation. Motivation and emotion. 1990;14:151-72.

24. Hatfield E, Cacioppo JT, Rapson RL. Emotional contagion. Current directions in psychological science. 1993;2(3):96-100.

25. Decety J, Jackson PL. The functional architecture of human empathy. Behavioral and cognitive neuroscience reviews. 2004;3(2):71-100.

26. Vreeke G-J, Van der Mark IL. Empathy, an integrative model. New Ideas in Psychology. 2003;21(3):177-207.

27. Zahn-Waxler C, Radke-Yarrow M, Wagner E, Chapman M. Development of concern for others. Developmental psychology. 1992;28(1):126.

28. Goodman R. The Strengths and Difficulties Questionnaire: a research note. Journal of child psychology and psychiatry. 1997;38(5):581-6.

29. Goodman R, Ford T, Simmons H, Gatward R, Meltzer H. Using the Strengths and Difficulties Questionnaire (SDQ) to screen for child psychiatric disorders in a community sample. The British journal of psychiatry. 2000;177(6):534-9.

30. Goodman R, Renfrew D, Mullick M. Predicting type of psychiatric disorder from Strengths and Difficulties Questionnaire (SDQ) scores in child mental health clinics in London and Dhaka. European child & adolescent psychiatry. 2000;9:129-34.

31. Bonthrone AF, Chew A, Kelly CJ, Almedom L, Simpson J, Victor S, et al. Cognitive function in toddlers with congenital heart disease: The impact of a stimulating home environment. Infancy. 2021;26(1):184-99.

32. Wolke D, Jaekel J, Hall J, Baumann N. Effects of sensitive parenting on the academic resilience of very preterm and very low birth weight adolescents. Journal of Adolescent Health. 2013;53(5):642-7.

33. Vanes LD, Hadaya L, Kanel D, Falconer S, Ball G, Batalle D, et al. Associations between neonatal brain structure, the home environment, and childhood outcomes following very preterm birth. Biological psychiatry global open science. 2021;1(2):146-55.

34. Bradley RH, Caldwell BM. 174 children: A study of the relationship between home environment and cognitive development during the first 5 years. Home environment and early cognitive development: Elsevier; 1984. p. 5-56.

**Appendix:**

**Cognitively Stimulating Parenting Scale (Age range: 1.5 to 6 years)**

| **Section 1: In this section,** please circle ‘YES’ or ‘NO’ **if the items correspond or not to the characteristics of your home environment:**  **In your home, does your child have access to:**  1. Toys that teach colours and shapes? Yes / No  2. Cassette/CD/DVD player? Yes / No  **In your home, does your child have access to:**  3. YouTube? Yes / No  4. Computers/iPads/iPhone? Yes / No  5. Learning apps, such as Peek-a-Boo, Peppa Pig or Fish School? Yes / No  6. Fine-motor toys such as LEGO, colouring books, or arts and crafts materials? Yes / No  7. Gross-motor toys such as trains, cars or bikes that your child can sit on and push along? Yes / No  8. A child-size table and chair? Yes / No  9. Toys that teach about household task such as sweeping, ironing, washing or other daily activities? Yes / No  10. Toys that stimulate number knowledge? Yes / No  11. A musical instrument that your child owns? Yes / No  12. 10 or more children’s books? Yes / No  **In your home, do you teach your child:**  13. Animal names? Yes / No  14. The alphabet? Yes / No  15. Colours? Yes / No  16. Shapes? Yes / No  17. Numbers? Yes / No  18. How shapes fit together? Yes / No  19. Words? Yes / No  20. Do parents read in their free time (or e-book)? Yes / No  21. Do parents read daily newspaper (or online)? Yes / No  22. Do parents regularly read magazines (or online)? Yes / No  23. Do parents follow current affairs? Yes / No  **Section 2: Please rate the below statements to indicate how often the activity typically occurs:**  Never Daily  1, Reading to child or telling stories 0 1 2 3 4 5 6  Less More  than 10 than 100  2, Number of books at home 0 1 2 3 4  Less than 2 per Daily  month  3, Frequency of family trips (i.e. zoo, 0 1 2 3 4 5  swimming, biking)  Never More than six per  year  4, Frequency of big trips or family holidays 0 1 2 3 4  Never More than six per  year  5, Frequency of museum visits 0 1 2 3 4 |
| --- |

**Scoring: To obtain single CSPS score, total scores from Sections 1 and 2**

Section 1: Yes = 1, No = 0,

Section 2: add the scores chosen by parent

|  |  |
| --- | --- |
